# A novel electrosurgical divider: performance in a self-controlled tonsillectomy study

**DOI:** 10.1007/s00405-021-07008-9

**Published:** 2021-08-02

**Authors:** Gerold Besser, Stefan Grasl, Elias L. Meyer, Julia Schnoell, Tina J. Bartosik, Faris F. Brkic, Gregor Heiduschka

**Affiliations:** 1 Department of Otorhinolaryngology, Head and Neck Surgery, Medical University of Vienna, Währinger Gürtel 18-20, 1090 Vienna, Austria; 2 Section for Medical Statistics, CeMSIIS, Medical University of Vienna, Vienna, Austria

**Keywords:** Haemorrhage, Pain, Palatine tonsil, Surgical device, Tonsillitis

## Abstract

**Purpose:**

Tonsillectomies are among the most common surgeries in otorhinolaryngology. A novel electrosurgical temperature-controlled instrument (device) promises rapid tonsillectomies and might reduce postoperative pain, but comparative studies to assess performance are warranted.

**Methods:**

This randomized self-controlled clinical trial was conducted from October 2019 to October 2020 at the Department of Otorhinolaryngology, Head and Neck Surgery of the Medical University of Vienna. Forty-eight patients underwent a tonsillectomy with the device on one side and using cold-steel with localized bipolar cauterization on the other side (control). Main outcomes were the time for tonsil removal (per side) and the time to stop bleeding (per side). Secondary measurements were postoperative pain, assessed once on day 0 and five times on days 1, 3, 5, 7, and 10. Postoperative bleeding episodes and consequences were recorded.

**Results:**

Device tonsillectomies were performed significantly faster than controls; the mean surgical time difference was 209 s (*p* < 0.001, 95% CI 129; 288). Intraoperative blood loss was significantly lower on the device side (all *p* < 0.05). Postoperative measurements of pain and bleeding were similar for both sides. Two return-to-theatre secondary bleeding events were recorded for the control side.

**Conclusion:**

The novel electrosurgical temperature-controlled divider reduced the tonsillectomy surgical time and intraoperative blood loss, with no apparent negative effects on postoperative pain or bleeding, compared to a cold-steel tonsillectomy with localized bipolar cauterization. In time-restricted settings, the device could be beneficial, particularly after familiarization with device handling.

**Trial registration:**

ClinicalTrials.gov Identifier: NCT03793816.

## Introduction

Tonsillectomies are among the most commonly performed otolaryngologic procedures and typically recommended when well documented, adequately treated sore throats occur at rates of 7 or more in 1 year, 5 or more per year for 2 consecutive years, or 3 or more per year for 3 consecutive years [1, 2].

Popular tonsillectomy techniques include a cold-steel dissection, combined with knot-tying or localized bipolar cauterization. Additionally, power surgical instruments are available. Frequent evaluations of surgical techniques are paramount for improving safety, patient care, and cost-effectiveness [3]. The most common morbidity is substantial postoperative pain, particularly in adults. Furthermore, the risk of secondary haemorrhage is well documented [4]. Temperature-controlled surgical instruments may cause less postoperative pain than conventional instruments, but reports on postoperative bleeding are inconsistent [5, 6].

The LigaSure™ is a bipolar device which measures tissue impedance to adjust the energy output. For tonsillectomies, favourable results have been suggested regarding postoperative pain [5, 7]. Lately, a similar device specifically for tonsillectomies with pistol-like grip and a long shaft (for narrow approaches) was released. It is indicated to seal vessels up to 3 mm in diameter, in contrast to the LigaSure™ sealing up to 7 mm. Both are powered by an energy platform which continuously measures impedance of clamped tissue and adjusts energy levels in real time. It automatically stops the energy delivery when the seal is complete (i.e., temperature-controlled). A pilot study on the novel device showed promising results in terms of surgical time [8]. However, the design and incomplete follow-up on postoperative pain impeded a proper interpretation of the associated morbidity.

This study tested the hypothesis that the tonsillectomy device would provide faster surgical times compared to the cold-steel technique with localized cauterization. To assess postoperative pain comprehensively, we planned a self-controlled, patient-blinded clinical trial.

## Materials and methods

### Ethical statement and registration

The study (registered at ClinicalTrials.gov Identifier: NCT03793816) was approved by the local Ethics Committee (EK No. 1399/2018) and conducted according to the guidelines of the Declaration of Helsinki on Biomedical Research Involving Human Subjects. All subjects provided written informed consent.

### Study design and participants

**Fig. 1:**
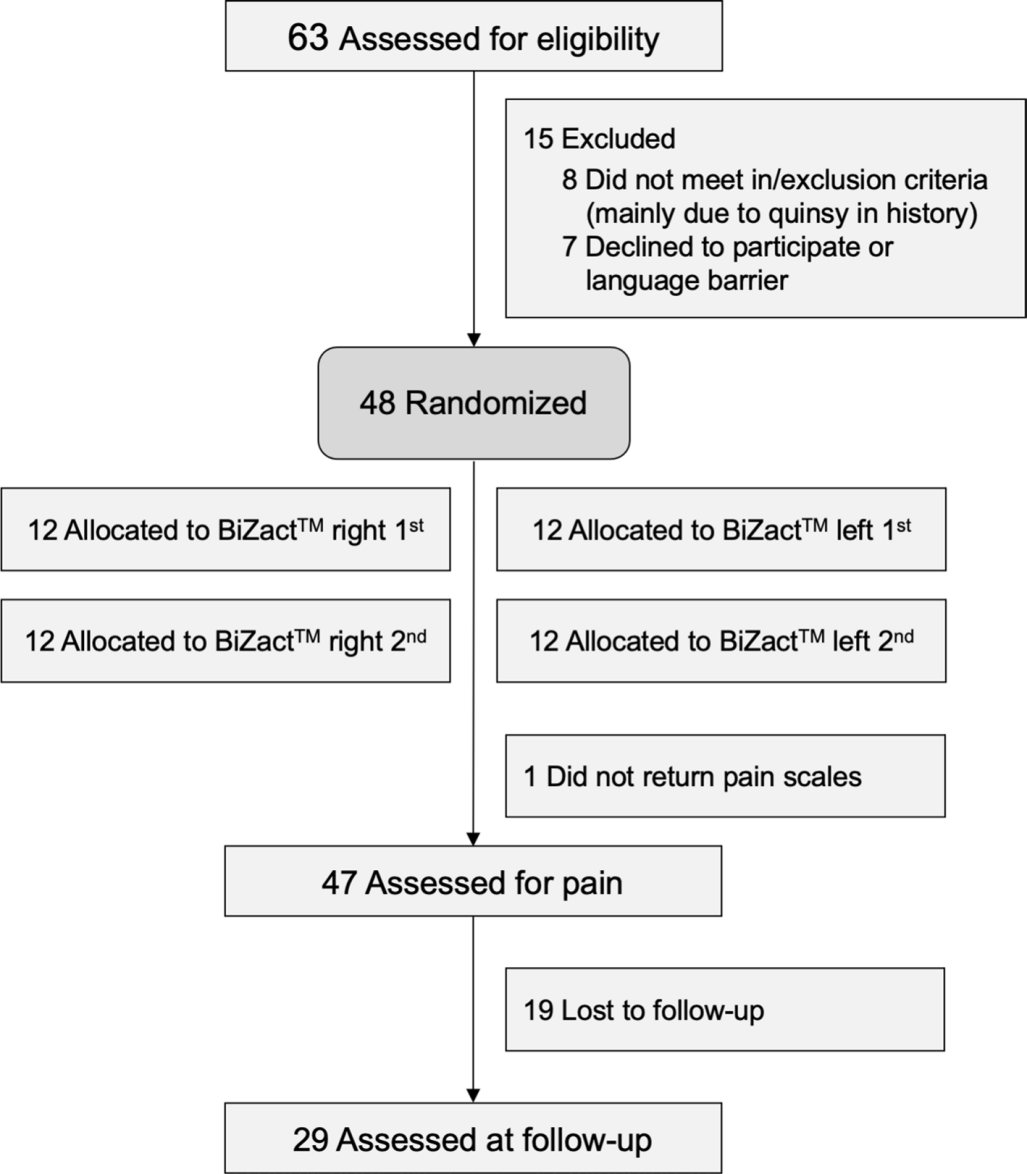
Study flowchart shows the patient selection process, tonsil randomization (left/right) procedure, and follow-up assessments

This study was a single-centre, randomized, self-controlled trial (RCT). The Consolidated Standards of Reporting Trials reporting guideline was followed. Each patient served as his/her own control [9]. Patients aged 14 years or older, with chronically recurrent tonsillitis and a planned tonsillectomy were eligible. Exclusion criteria: a history of abscesses in the tonsillar region (i.e., quinsy), a coagulation disorder, suspicion of an untreated malignancy, and planned removal of only one tonsil. Figure 1 depicts the study flow diagram.

### Procedures, randomization, and blinding

For electrosurgical removal of one randomised tonsil, we used the BiZact™ Open Sealer/Divider (Medtronic/Covidien IIc, Mansfield, MA, USA), a single-use device (here, referred to as “device”), connected to a Valleylab™ FT10 Energy Platform. It creates a seal by applying radiofrequency energy to tissue interposed between the jaws. A cutting blade is then surgeon-activated. We performed an incision of the palatal arch, located the cranial pole of the tonsil, dissected along the tonsil capsule, and detached the tonsil.

The contralateral tonsil was removed with a cold-steel technique (i.e., scissors and/or Freer raspatories), with localized bipolar forceps for cauterization, and compression with cotton balls (here, referred to as the “CSB” or the control side). The analgesic regime included non-steroidal anti-inflammatory drugs, and occasionally, tramadol.

Block randomization (block size of 12) was applied to determine the side to begin with (first side, left/right) and the technique to begin with (the device or the CSB). Patients were blinded to the sidedness, and un-blinding was planned with a follow-up phone call (see below).

### Outcomes

The main outcome measurement was the time from the first incision to tonsil placement on the instrument table. Surgeon handedness was recorded, and experience was classified, based on the number of tonsillectomies performed prior to trial participation (less than 10 times, between 10 and 20 times, between 20 and 30 times, and more than 30 times).

Secondary intraoperative outcomes were the time required to stop bleeding, and blood loss, measured as the suction fluid volume including flush water and the number and weight of cotton balls used (scale, KERN & SOHN GmbH, Balingen, Germany).

Postoperative outcomes were paper–pencil ratings of pain on a visual analogue scale (VAS) of 100 mm for each side. Pain was evaluated once after awakening from surgery (day 0) and 5 times per day (at waking up, during breakfast, before lunch, during dinner, and before falling asleep) on days 1, 3, 5, 7, and 10. Patients re-visited the clinic once to return pain scales, discuss histological findings, and undergo a wound healing assessment. Wound healing was rated on a VAS of 100 mm for the right and left tonsillar fossae (from 0 mm =“no coatings/inflammation”, to 100 mm =“massive coating/inflammation”).

Bleeding events were categorized as follows: type I: no intervention required; type II: bleeding controlled with local measures; type III: bleeding controlled in the operating room; i.e., a return-to-theatre (RTT) event; IV: external carotid artery ligation required; and V: death [10].

Approximately 2 months later, a follow-up phone call was planned. Patients were interviewed with yes/no questions on residual pain and side differences.

### Statistical analysis

The sample size calculation was based on previous experience with the device and the average time for performing a CSB. A sample size of 42 was calculated to have 80% power for detecting a difference of 2 min between mean times, assuming a standard deviation of 4.5 min, when evaluated with a paired *t* test and a 0.05 two-sided significance level. To provide for potential dropouts, 48 patients were included in this study.

To assess the primary objective, we performed a linear mixed model regression with the surgical time as a dependent variable, the surgical device (device vs. CSB) and sequence (first or second tonsil) as fixed effects, a random intercept for each patient and each surgeon. Also, the effects of other variables on the surgical time were evaluated. In all cases, we used the same mixed model, but we added each variable of interest as a further fixed effect to the model. For the secondary objectives, the dependent variable was the time to stop bleeding.

In regard to pain difference, for each time point, the mean difference in pain between the control and device sides was computed, with the 95% confidence interval (95% CI).

*p* values < 0.05 were considered statistically significant. These *p* values and corresponding 95% CIs were for descriptive purposes only; hence, no multiplicity corrections were applied. All analyses and data visualizations were performed with R version 3.6.3 (R Foundation for Statistical Computing, Vienna, Austria).

## Results

Between Oct 1, 2019, and Oct 23, 2020, 63 patients (14 years of age and older) were scheduled for a bilateral tonsillectomy. Fifteen patients were excluded, because they did not fulfil the eligibility criteria or were not willing to participate. As intended, 48 subjects (37 females, mean age 24.3 ± 6.7 years, range 16–40 years) were included. Baseline patient characteristics and surgical parameters are shown in Table 1. No adverse device-related events or serious adverse events were reported.

**Table 1:** Patient demographics and intraoperative parameters for the two tonsillectomy approaches

Parameters	*n* = 48, Age 24.3 ± 6.7 years, (37 females (77%))	
	BiZact™, n = 48	Control, *n* = 48
Surgical time (s)	307.5 ± 162.4	516.1 ± 300.8
Stop bleeding time (s)	72.4 ± 151.7	177.0 ± 189.4
Suction volume including flush water (ml)	43.1 ± 44.5^a^	132.2 ± 193.4
Cotton balls, number	1.6 ± 0.8	2.5 ± 1.5
Cotton balls, weight (mg)	2.7 ± 2.6	6.8 ± 7.2

Values are the mean ± standard deviation

^a^In 24 cases of the device side, suction volume was not measurable accurately; most surgeons recorded these volumes as “below the first mark of 50 ml”

### Intraoperative findings

**Fig. 2:**
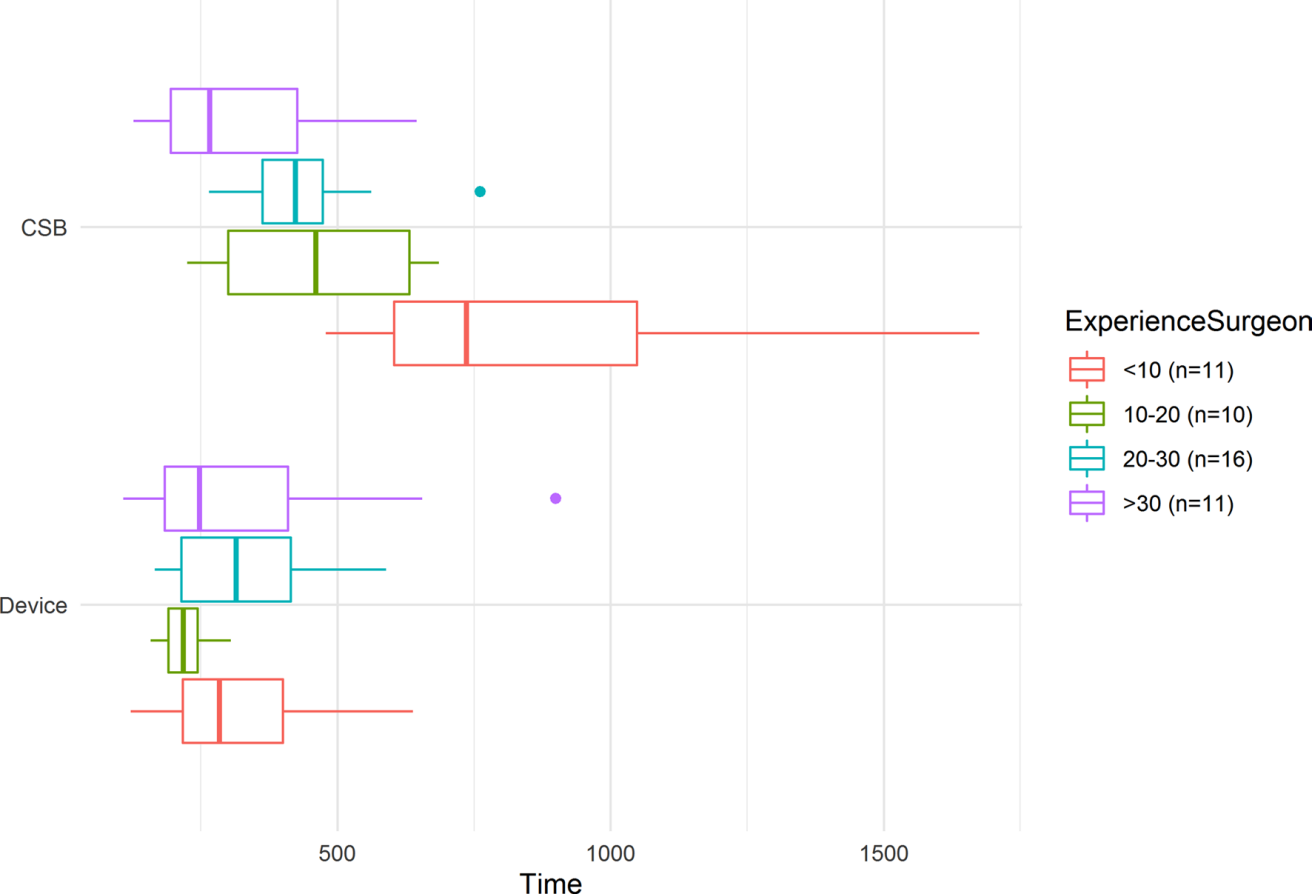
Tonsillectomy surgical times (s) for the BiZact device side (Device) and for the control cold-steel side (CSB), grouped by surgeon experience (e.g., less than 10 tonsillectomies in their careers, etc.)

Device tonsillectomies required significantly less time than controls. The mean time difference was 209 s (95% CI 129; 288; *p* < 0.001). Previous experience was significantly associated with shorter surgical times (*p* = 0.015). Figure 2 shows the surgical time required for each technique, grouped by surgical experience. In 15 patients, the device side was completed more than 5 min faster than the CSB side. Additionally, significantly less time was required to stop bleeding on the device side than on the control side (estimated difference = 105 s, 95% CI 57; 153; *p* < 0.001). When we included the time to stop bleeding in the absolute surgical time per side, the device side was completed more than 5 min faster in 22 cases, and 10 min faster in 9 cases, compared to the control side. For both 5 and 10 min, these magnitudes were never observed for the control side. For included under-aged patients (*n* = 8), the mean time difference was 346 s, for adults 170 s (*n* = 40).

With each use of the device by the same surgeon, the mean surgical time was significantly reduced (estimated reduction per use = 36 s, 95% CI 3; 69; *p* = 0.035), which reflected a significant learning effect.

Intraoperative blood loss was significantly lower on the device side. The suction fluid volume difference was estimated at 89 ml (95% CI 36; 142; *p* = 0.002), and the cotton ball weight difference was estimated at 4.1 mg (95% CI 2.2; 6.0; *p* < 0.001).

### Postoperative pain ratings

**Fig. 3:**
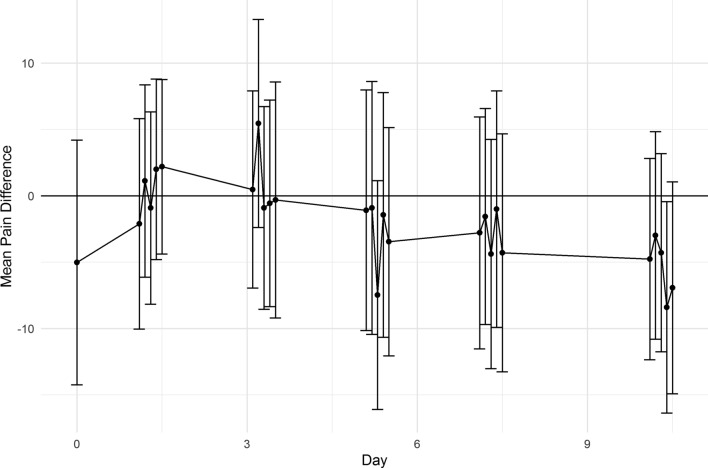
Mean postoperative pain differences between cold-steel (control) and BiZact device sides. Pain was assessed once on day 0, and 5 times per day on several postoperative days (total 26 time points). Symbols indicate the mean difference between pain ratings on the control and device sides (i.e., VAS of device pain–VAS of control pain), and whiskers indicate the 95% confidence intervals. Negative values indicate greater pain on the control side, and positive values indicate greater pain on the device side. VAS: visual analogue scale

Out of 48 patients, 47 (97.9%) returned the paper–pencil VAS ratings on pain for each pharynx side. The mean differences in pain are depicted in Fig. 3. At most time points (21/26, 80.8%), pain was greater on the control side, however, these differences were minimal. For all time points, the absolute mean pain difference was below 10 mm (i.e., no left–right pain differences more than 10 mm), and at most time points (21/26, 80.8%), the mean pain difference was below 5 mm on the VAS. With only one exception (fourth measurement on day 10), all 95% CIs included the value 0, which indicated no significant differences in pain between the two surgical approaches. These pain findings were seen also in the underage group and adult group alone likewise.

### Bleeding

We did not observe any primary bleeding events (i.e., within 24 h after surgery). Secondary bleeding (i.e., more than 24 h after surgery) occurred at a mean of 5.6 ± 1.7 days after surgery. Among these, in 4 patients (*n* = 2 device side, *n* = 2 control side), a type I bleeding was recorded. In addition, one patient reported a minor bleeding event that occurred at home, but the side was not specified. In another 6 patients (*n* = 4 device side, *n* = 2 control side), a type II bleeding was recorded. In one patient (control side), a type III (RTT) bleeding was recorded. Additionally, in 4 patients, a second bleeding event was recorded: 3 were a type II bleeding (*n* = 2 device side, *n* = 1 control side), and one was a type III (RTT) bleeding (control side).

### Wound healing and follow-up

Wound healing was rated in 27 cases at a mean of 16.0 ± 17.6 days after surgery with no significant differences between the 2 techniques (*p* > 0.05). Twenty-nine patients were reached at a mean of 83.9 ± 21.0 days after surgery for follow-up. One patient reported residual pain. Nine patients reported that swallowing felt different on the two sides; it was less comfortable on the device side in 3 cases and the control side in 6 cases.

## Discussion

Clinicians must be aware of potentially delicate consequences following tonsillectomies, such as severe pain or even life-threatening bleeding. The latest indication criteria should be followed, because evidence remains sparse on the benefits of tonsillectomies in adults [3, 11]. Of note, a recent study (> 1 million patients) showed that a paediatric tonsillectomy could increase the likelihood of later developing respiratory, infectious, and allergic diseases [12]. Those data are currently unavailable for adults, but in the meantime, well thought-through studies on tonsillectomy techniques are valuable. Surgeries that target paired organs on both sides of the body provide the potential for self-controlled trails. With regard to side-dependent pain assessments, this approach elegantly facilitated a blinded strategy, which has only been applied in few tonsillectomy studies to date [13, 14].

In this RCT, we found that a novel electrosurgical divider could reduce the tonsillectomy operating time, compared to the cold-steel tonsillectomy with bipolar localized cauterization. Our results suggested that it could potentially save up to 20 min of the time required for both sided tonsil removal and bleeding control. In selective health care settings, this might improve the cost-effectiveness of the device. Our data show this can particularly be the case when the device is in the hands of surgeons with fewer tonsillectomies performed in their career. In addition, intraoperative blood loss was significantly lower on the device side, which could be relevant in paediatric populations (due to less total blood volume). However, postoperative measurements, such as pain and bleeding, were rather similar for the device and control sides.

Pain is an important issue in adult tonsillectomies (as opposed to paediatric tonsillectomies) and associated with cost-intensive analgesic consumption and potential readmissions [11]. A potential explanation could be that adults tend to have more scar tissues, due to previous infections, and consequently, they require more “invasive” surgical techniques with “hot” cauterization [13]. Previous studies found that postoperative pain was reduced when temperature-controlled instruments were used [6]. An internal report conducted at Medtronic stated that the device generated less external heat compared to a monopolar electrosurgical device (79.6 ± 2.4 °C versus 123.9 ± 10.0 °C) [15]. Based on this and findings with a similar technique [5, 7], we expected to observe less postoperative pain on the device side. Of note, we only observed a slight trend for less pain on the device side, starting on day 3 (Fig. 3). This might be explained by the control technique: localized electrocautery might have produced less thermal tissue damage, and hence less pain, compared to broader electro-cauterization. Therefore, our control technique might have precluded a clear difference in pain. Further studies are warranted to compare the device to “fully hot” techniques, such as monopolar devices and continuous electrocautery. Also, comparative studies including plasma coblation are warranted which has been shown to be superior to electrocautery in terms of postoperative pain [16].

Overall, we encountered high rates of secondary bleeding episodes: an initial event occurred in 25% (12/48 patients), and a second event occurred in 8.3% (4/48 patients). These events were nearly equally distributed between the device and control sides. Various considerations should be highlighted in this context. First, previous studies have reported variable rates of secondary bleeding; moreover, actual bleeding rates can be much higher than recorded [4, 17, 18]. Second, after reports of fatal bleeding episodes in children in Austria in 2007, the local professional ear, nose, and throat (ENT) community has become highly sensitized and alert [19]. Patients are repeatedly advised to revisit the hospital immediately when bleeding occurs at home, even minor bleeding. Frequently, patients revisit with only blood stains in their sputum and are typically readmitted every time. With this in mind, it might be more meaningful to pay attention to type III bleeding events, in terms of RTT events. In the present study, 2 type III events (4.2%) occurred on the control side (none on the device side). This percentage was lower than the rates reported in two larger studies, which showed 5.6% and 9.2% RTT events [4, 20]. Currently, the use of this device has increased in the ENT community, which will generate larger data sets. Therefore, in future, we may be able to gain more profound insight on secondary bleeding events.

This study had some limitations. Although this trial was not impacted by financial support or personal interests [21, 22], the novelty of the device at our clinic may have influenced surgeon enthusiasm. The challenge of performing two techniques in one patient could have encouraged the surgeons to operate faster than usual. However, this argument should not be over-interpreted as the procedure quickly became routine. Another limitation was that the fluids used to flush the suction system were not recorded and only the marks on the canister were used to estimate blood loss. In half of the cases, the device produced very little blood, and the investigators either recorded it as “below the first benchmark” (i.e., less than 50 ml) or “not measurable”. Consequently, fewer actual values were available for analysis. Finally, larger sample sizes in future studies on the device are needed to comprehensively evaluate postoperative pain and bleeding events.

## Conclusion

This self-controlled randomized clinical trial showed that the novel temperature-controlled electrosurgical divider could reduce tonsillectomy operative times and intraoperative blood loss, with no apparent negative effects on postoperative pain or bleeding, compared to a cold-steel tonsillectomy with localized bipolar cauterization. In time-restricted settings, the device might provide a benefit once surgeons have become accustomed to handling the device.
